# Upregulation of long non-coding RNA ENSG00000267838 is related to the high risk of progression and non-response to chemoradiotherapy treatment for cervical cancer

**DOI:** 10.1016/j.ncrna.2024.10.004

**Published:** 2024-10-24

**Authors:** Bruna Custódio Dias Duarte, Fábio Ribeiro Queiroz, Álvaro Percínio Costa, Angelo Borges de Melo Neto, Carolina Pereira de Souza Melo, Paulo Guilherme de Oliveira Salles, Wander de Jesus Jeremias, Pedro Luiz Lima Bertarini, Laurence Rodrigues do Amaral, Letícia da Conceição Braga, Matheus de Souza Gomes, Agnaldo Lopes da Silva Filho

**Affiliations:** a Laboratório de Bioinformática e Análises Moleculares, Universidade Federal de Uberlândia, 38702-178, Patos de Minas, MG, Brazil; b Laboratório de Pesquisa Translacional Em Oncologia, Instituto Mário Penna, 30380-490, Belo Horizonte, MG, Brazil; c Laboratório de Farmacologia Experimental, Escola de Farmácia, Universidade Federal de Ouro Preto, 35402-163, Ouro Preto, MG, Brazil; d Programa de Pós-graduação Em Ciências Aplicadas à Cirurgia e à Oftalmologia, Faculdade de Medicina, Universidade Federal de Minas Gerais, 31.270-901, Belo Horizonte, MG, Brazil

**Keywords:** Gene expression, Regulatory profile, Biomarkers, Non-coding RNA, Cervical cancer, Prognosis

## Abstract

Cervical cancer (CC) is a global public health concern, primarily caused by persistent infection with oncogenic types of human papillomavirus (HPV). The World Health Organization (WHO) has established a plan to eliminate CC as a public health issue by the year 2100. However, the implementation of the HPV vaccine is impeded by vaccine restrictions and misinformation despite its demonstrated effectiveness. The CC treatment is influenced by the disease stage, with an unfavorable prognosis for those in advanced stages. This study aimed to investigate the potential of long non-coding RNAs (lncRNAs) in CC by identifying and characterizing related lncRNAs, elucidating their regulatory mechanisms and molecular interactions, and analyzing their expression patterns in patients with diverse responses to chemoradiotherapy. Non-stem cells from CC were isolated using flow cytometry sorting and used for total RNA extraction. The RNA was used to build libraries that were subsequently sequenced using the Illumina Nextseq 550.417 lncRNAs that showed differentially expressed between CC patients who responded or not to treatment. Further analysis demonstrated that these lncRNAs significantly interact with several molecules, which play crucial roles in CC progression and therapeutic resistance. Statistical analysis correlated the expression profile of these lncRNAs with treatment efficacy. Three lncRNAs, *ENSG00000267838*, *ENSG00000266340*, and *FRMD6-AS1*, were identified with positive expression related to non-response to chemoradiotherapy and worse progression-free survival in CC patients. Specifically, lncRNA *ENSG00000267838* has its up-regulation related to non-response and down-regulation to response to chemoradiotherapy treatment.

## Highlights

• 417 differentially expressed lncRNAs were identified in the cervical cancer samples.
• A pattern of expression was identified for response and non-response to treatment.
• The lncRNAs ENSG00000266340 and FRMD6-AS1 are correlated with worse prognosis in CC.
• ENSG00000267838 affects the high risk of progression and non-response to treatment.
• Potential of lncRNA ENSG00000267838 as a discriminative marker for R and NR groups.

## 1 Introduction

Cervical cancer (CC) is a preventable disease and is the fourth most common cancer in women worldwide. In 2020, there were approximately 604,000 new cases and 342,000 deaths in low- and middle-income countries, according to a World Health Organization (WHO) report [1]. The most common histologic forms of CC include squamous cell carcinoma (SCC) in 75–95 % of cases and adenocarcinoma (ADC) in 10–25 % of cases [2]. CC, primarily caused by HPV types 16 and 18, involves E6 and E7 proteins that deactivate tumor suppressor genes, such as TP53 and Rb. The WHO has set the goal of eliminating CC as a public health problem by 2100 through the 90-70-90 global strategy, which must be adopted by the year 2030 [3,4]. Early diagnosis significantly improves the 5-year survival rate, ranging from 92 % to 17 % in metastatic cases, emphasizing the impact of the diagnosis stage on patient prognosis [5].

Despite progress in vaccination and screening, the gradual decline in CC incidence persists [6]. The primary therapeutic approach involves cisplatin-based chemoradiotherapy, often combined with paclitaxel, yielding response rates of 29.1 %–67 % [7]. However, intrinsic or acquired cisplatin resistance poses a significant challenge influenced by processes such as epithelial-mesenchymal transition (EMT), DNA methylation changes, miRNA profiling, cancer stem cell characteristics, and stress response chaperone expression [8].

Integrating biomarkers into CC screening is challenging because the lack of a uniform scoring system hinders the consistent application of techniques such as immunohistochemistry and cytology [7]. Biomarker discovery studies aim to improve CC detection and post-treatment monitoring by searching for novel biomarkers. Long non-coding RNAs (lncRNAs) are characterized by differential expression, suggesting involvement in processes such as cell growth, differentiation, migration, invasion, and apoptosis [9].

The specific expression patterns of lncRNAs play a fundamental role as biomarkers in different stages of cancer. In prevention, the detection of lncRNAs in body fluids such as blood, serum, and urine allows the prescription of specific suppressors or activators of these lncRNAs to patients [10]. In diagnostics, identifying the presence or absence of lncRNAs in patient samples provides a precise tool for distinguishing different types of cancer. In prognosis, lncRNAs can be used to correlate the patient's cell proliferation, metastasis, and apoptosis status using samples of cancer cell mass, urine, and blood [11]. In the therapeutic approach, specific methods are being developed to target lncRNAs, providing innovative perspectives and therapeutic strategies [10].

Another unique feature of lncRNAs as biomarkers is their highly tissue-specific expression. This, together with the remarkable stability during circulation in body fluids, especially in exosomes or apoptotic bodies, makes lncRNAs resistant to the degradation activities of ribonucleases [12]. Furthermore, lncRNA expression's deregulation in tumor tissues is detectable in body fluids, including saliva and gastric fluid. Compared to conventional methods, the use of lncRNAs is minimally invasive and better tolerated by patients. In the cancer scenario, these biomarkers can distinguish patients with early-stage tumors from healthy individuals with high sensitivity and specificity. In addition, lncRNAs offer the prospect of predicting the prognosis of patients with tumors and assessing the risk of tumor metastasis and recurrence after surgery, allowing a comprehensive analysis of the success of surgical intervention [12,13].

The role of lncRNAs in cancer is closely related to their expression profile and ability to interact with proteins, mRNAs, and miRNAs. In the case of CC, resistance to antineoplastic drugs is a limitation in chemoradiotherapy treatment. Examples of the lncRNA roles in cervical cancer include the *PVT1* lncRNA, which is associated with resistance to cisplatin and paclitaxel, as well as promoting EMT. Another example is the *CASC2* lncRNA, whose low expression contributes to resistance to cisplatin, while the *PTEN* lncRNA can increase sensitivity to chemotherapy in CC. Additionally, high expression of *NEAT1* lncRNAs correlates with resistance to radiotherapy in CC [9,14].

This study aims to fill a critical gap in understanding CC by focusing on CC non-stem cells (CCNSCs). Identifying lncRNA expression profiles linked to therapeutic response in CC presents a promising opportunity for discovering biomarkers that can be accessed through liquid biopsy, both for diagnosis and monitoring treatment efficacy. This approach can potentially improve the speed and effectiveness of treatment, enabling the implementation of more personalized medicine that can substantially influence survival outcomes.

## 2 Patients and methods

**Fig. 1:**
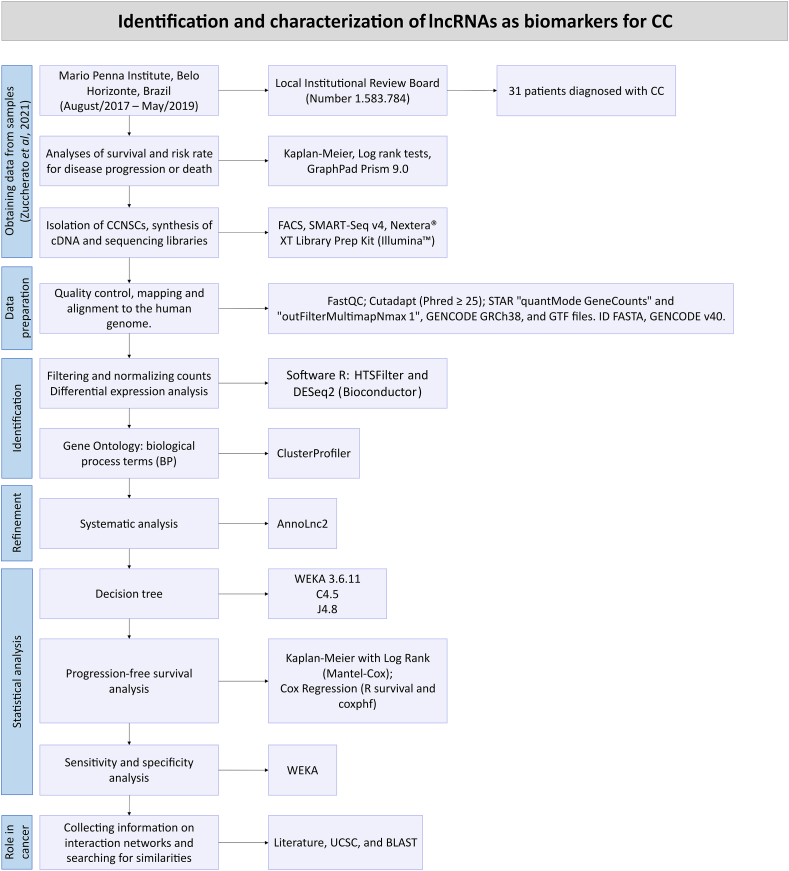
**– Pipeline describing the steps to identify and characterize lncRNAs associated with response to chemoradiotherapy treatment for CC patients.** The steps are divided into the following categories: obtaining data from patient samples; sample preparation; identification of DEGs; refinement of DEGs; statistical analysis; and investigation of the role of these lncRNAs in cancer.

Samples were collected from 31 patients diagnosed with CC at Instituto Mario Penna (Belo Horizonte, MG Brazil), between August 2017 and May 2019, as first described by Zuccherato et al. [15]. Inclusion criteria were no history of cancer or immunologic disease, histopathologic diagnosis of adenocarcinoma or squamous cell carcinoma, and signed informed consent. This study was approved by the local Institutional Review Board (approval number 1.583.784) [15]. A systematic assessment of molecular interactions and gene expression in response to treatment in our cohort was done as shown in the pipeline presented in Fig. 1.

### 2.1 Sequencing libraries preparation

Fluorescence-activated cell sorting (FACS) was used to isolate cervical cancer stem cells (CCSCs) and cervical cancer non-stem cells (CCNSCs) from a complex mixture of tumor cells based on their light scattering and fluorescence staining profile, as described by Zuccherato et al. [15]. The complete methodology, including casuistry, RNA preparation steps, and subsequent sequencing, is available in the article by Ref. [15,15]). Sixty-two libraries with more than 20 million reads each were analyzed. Only sequences related to the CCNSCs portion of RNAseq were analyzed in this study.

### 2.2 Data preparation

For the sequence quality analysis, the FastQ files containing the sequences were evaluated using the FastQC program (https://www.bioinformatics.babraham.ac.uk/projects/fastqc/). The Cutadapt tool [16] was used to remove low-quality sequences with a Phred score ≤25 at the 3′ end of the reads and adaptors. The integrity of the sequences was then re-assessed using FastQC.

STAR software (https://github.com/alexdobin/STAR) was used to map and align the clean sequences to the human genome (*Homo sapiens* GRCh38). The parameters “quantMode GeneCounts” and “outFilterMultimapNmax 1″, based on the GRCh38 and GTF files of the reference human genome obtained from the GENCODE database, were used to quantify the transcripts that aligned exclusively to a specific gene. LncRNAs genes with counts less than ten were disregarded. The FASTA sequences of the obtained IDs were retrieved from the nucleotide sequence file of lncRNA transcripts in the reference chromosomes, version 40, from GENCODE (https://www.gencodegenes.org/human/). The sequences are available in the SRA database (PRJNA812529).

Differentially expressed genes (DEGs) were visualized on heat maps using the Pheatmap package in R (https://www.rdocumentation.org/packages/pheatmap/versions/1.0.12/topics/pheatmap). For analysis, only DEGs with a log2FC > 1 or < −1 and an adjusted p-value (padj) < 0.05 were considered. These DEGs were utilized as targets in ClusterProfiler [17] to explore their biological functions.

### 2.3 Analysis of the functional roles of lncRNAs

The AnnoLnc2 program [18] was utilized for the systematic annotation of lncRNAs, enabling a comprehensive functional study, including expression patterns, co-expression analysis in normal and cancer tissues, regulation, interactions with biomolecules, genetic associations, and evolution. To understand the biomolecules associated with the transcripts, literature searches, and database queries were conducted using Human Protein Atlas - HPA (https://www.proteinatlas.org/), Genotype-Tissue Expression - GTEx (https://www.gtexportal.org/home/), Universal Protein - UniProt (https://www.uniprot.org/), BLAST (https://blast.ncbi.nlm.nih.gov/Blast.cgi), and UCSC (University of California at Santa Cruz) Genome Browser (https://genome.ucsc.edu/) databases.

### 2.4 Decision tree analysis for DEGs

The C4.5 algorithm from WEKA software (version 3.6.11, Waikato Environment for Knowledge Analysis, University of Waikato, New Zealand) was implemented using the parameters of the J4.8 algorithm [19,20] to construct a decision tree from a dataset consisting of 31 records, divided into two groups: Responder (R) and Non-responder (NR). The entire dataset (FULL) was used to develop and train the classification algorithms for classification. The performance of the classification algorithms was estimated using leave-one-out cross-validation (LOOCV). Sensitivity and specificity analyses were conducted using WEKA software.

### 2.5 Survival and risk analysis

Kaplan-Meier (log-rank) analyses were performed using SPSS software (version 20, IBM, USA) to evaluate an association between the expression profile of DEGs and progression-free survival. Cox regression analysis was used to quantify the impact of this association. The R survival and coxphf packages (https://cran.r-project.org/web/packages/) were used. The hazard ratio (HR) values obtained were visualized in a forest plot generated in GraphPad Prism 9 (GraphPad Software, USA). Only results with a p-value <0.05 were considered statistically significant.

## 3 Results

### 3.1 Clinical characteristics of patients

The clinicopathological characteristics of the 31 patients who were treated with chemoradiotherapy for CC are provided in Table 1.

**Table 1:** Histopathologic characteristics of the 31 patients included in the study.

CHARACTERISTICS	N	%
**DIAGNOSIS**		
Adenocarcinoma	1	3,23
Squamous cell carcinoma	30	96,77
**HISTOLOGICAL GRADE**		
II	19	61,29
III	12	38,71
**FIGO CLASSIFICATION**		
IIA	1	3,23
IIB	13	41,93
IIIB	17	54,84
**PARAMETRIAL INVOLVEMENT**		
Unilateral	9	29,03
Bilateral	21	67,74
Free	1	3,23
**VAGINAL INVOLVEMENT**		
Present	27	87,10
Absent	2	6,45
Not applicable	2	6,45
**ANTICANCER AGENT**		
Cisplatin	30	96,77
Carboplatin	1	3,23
**STATUS AFTER 8 MONTHS OF TREATMENT**		
Responder	21	67,74
Non-Responder	10	32,26
**METASTASIS**		
After 8 months of treatment		
Present	9	29,03
Absent	18	58,07
Not applicable	4	12,90

N, number of patients.

### 3.2 Differential expression profile analysis

**Fig. 2:**
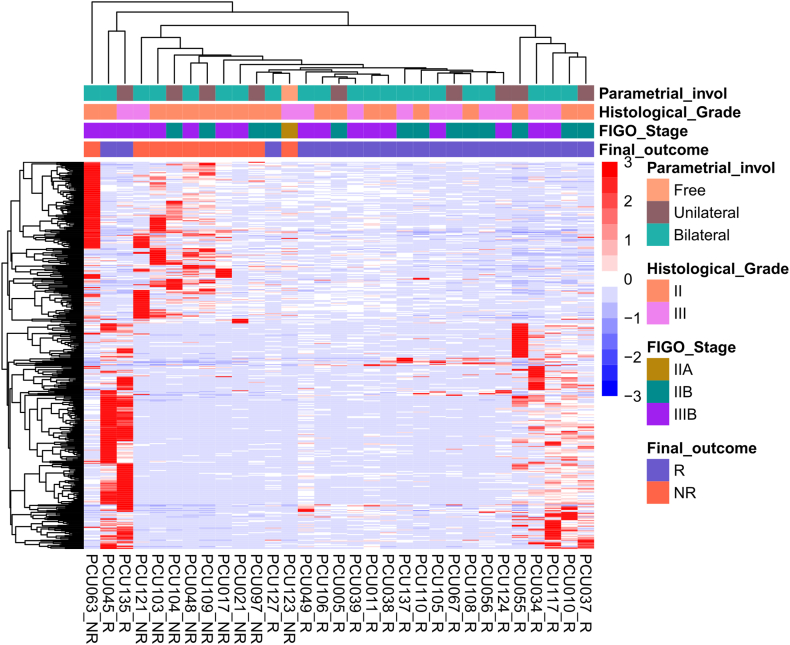
**– Heatmap representing 417 DEGs comparing patients 21 R to 10 NR CC patients.** LogFoldChange >1 or < −1 and padj value < 0.05 were considered. Indicators: “Parametrial involvement” represents parametrial involvement as free (salmon), unilateral (brown) or bilateral (aqua green). “Histologic grade” is histologic grade II (light pink) or III (dark pink). “FIGO stage” shows which FIGO grade the patient is in, IIA (gold), IIB (dark green) or IIIB (light purple). “Final outcome” is the treatment status, R (purplish-blue) or NR (reddish orange).

In the comparative analysis between the R and NR groups, 417 out of 18,805 lncRNAs annotated in GENCODE version 40 were identified as differentially expressed genes. This variance in gene expression profiles between the groups indicates a potential link between lncRNA expression and the response to treatment in CC. The investigation then examined the expression profiles of these differentially expressed lncRNAs in CCNSCs derived from both R and NR groups. Distinct gene signatures were observed between R and NR patients, suggesting that the expression profile may be associated with treatment response in CC (Fig. 2).

### 3.3 Gene Ontology analysis

Only transcripts that showed significant enrichment in Gene Ontology (GO) terms, with an adjusted p-value of less than 0.05, were used to elucidate the role of lncRNAs in treatment outcomes. Out of the 30,065 transcripts categorized under the biological process (BP) category, 18,505 were lncRNA genes in the background list, and 417 were differentially expressed genes in the target list for this analysis. This category highlighted five lncRNA genes: *SNHG29*, *CDKN2B-AS1*, *NEAT1*, *MALAT1*, and *SCARNA9*.

### 3.4 Systematic analysis of DEGs

The comprehensive examination of the 417 DEGs through the AnnoLnc2 software enabled an in-depth understanding of the roles the studied lncRNAs may play. This analysis encompassed a variety of factors, including the potential for coding, genomic positioning, secondary structural characteristics, and expression data. Additionally, it investigates co-expression patterns, subcellular localization, evolutionary history, interactions with proteins and miRNAs, the presence of repeat elements, and the regulation of gene transcription. By expanding the scope of the investigation to include the entire female reproductive system beyond just cervical tissue, the study discovered that 28 of the 417 DEGs are expressed in normal vaginal tissue, 21 in the ovary, and 19 in the uterus.

**Fig. 3:**
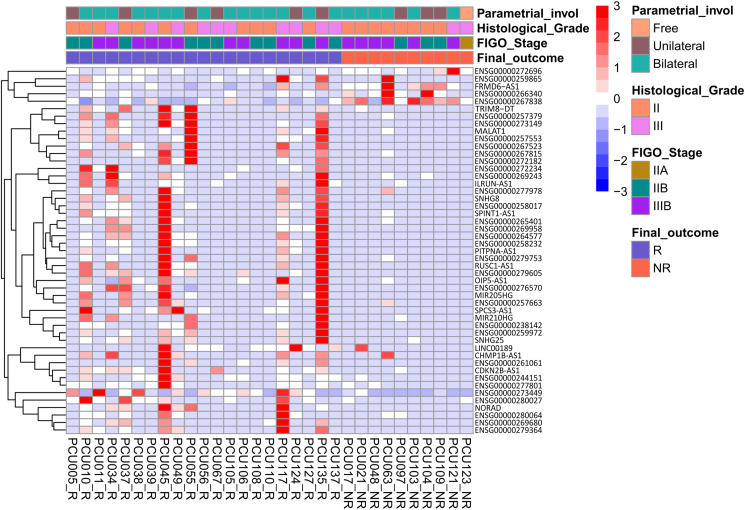
**– Heatmap representing 49 DEGs associated with CC.** Comparison between 21 R and 10 NR patients. LogFoldChange >1 or < −1 and padj value < 0.05 were considered. Positively regulated DEGs are shown in red, negatively regulated DEGs in blue and those with constant expression in white. Light red indicates DEGs with a z score <1, while light blue indicates DEGs with a z score > −1. The intense red and blue colors represent DEGs with a z score >1 or < −1.

The AnnoLnc2 software was utilized to sift through the differentially expressed lncRNA genes to pinpoint transcripts of potential significance to the biological processes implicated in CC. The initial step involved assessing candidates for their expression across a range of tissues, including the endocervix, ectocervix, uterus, ovaries, and vagina, in addition to cell lines (notably HeLa) and cancers affecting the female reproductive system, with an emphasis on CC. This analysis stage identified 49 DEGs that satisfied these criteria, as detailed in Supplementary Table 1. Subsequently, the expression profiles of these 49 transcripts were examined, revealing a distinct gene signature capable of distinguishing between the R and NR groups (Fig. 3).

### 3.5 Refinement of the 49 lncRNAs

A meticulous refinement strategy was employed in our quest to identify transcripts of the potential relevance of 49 lncRNAs in CC. This process involved the analysis of transcript interactions with various biomolecules, prioritizing significant interactions and punctuating the biological functions executed by these molecules. The analysis of interactions of 49 lncRNAs with biomolecules, such as TFs, proteins, and miRNAs, performed by AnnoLnc2, aimed to evaluate associations relevant in the context of CC (Supplementary Table 3). Our analyses have uncovered a pivotal and multifaceted role for miRNAs associated with lncRNAs in the CC scenario, as outlined in Supplementary Table 4. Through this rigorous refinement process, a subset of 8 DEGs was identified as particularly pertinent: *MIR205HG*, *NORAD*, LINC00189, *RUSC1-AS1*, *PITPNA-AS1*, *CDKN2B-AS1*, *MIR210HG*, and *OIP5-AS1*. This selection was carefully made with an emphasis on the interactions of these lncRNA genes with transcription factors (TFs), miRNAs, and proteins previously linked to CC. The aim was to point out promising candidates that merit further exploration.

### 3.6 Analysis of DEGs selected as potential biomarkers

**Fig. 4:**
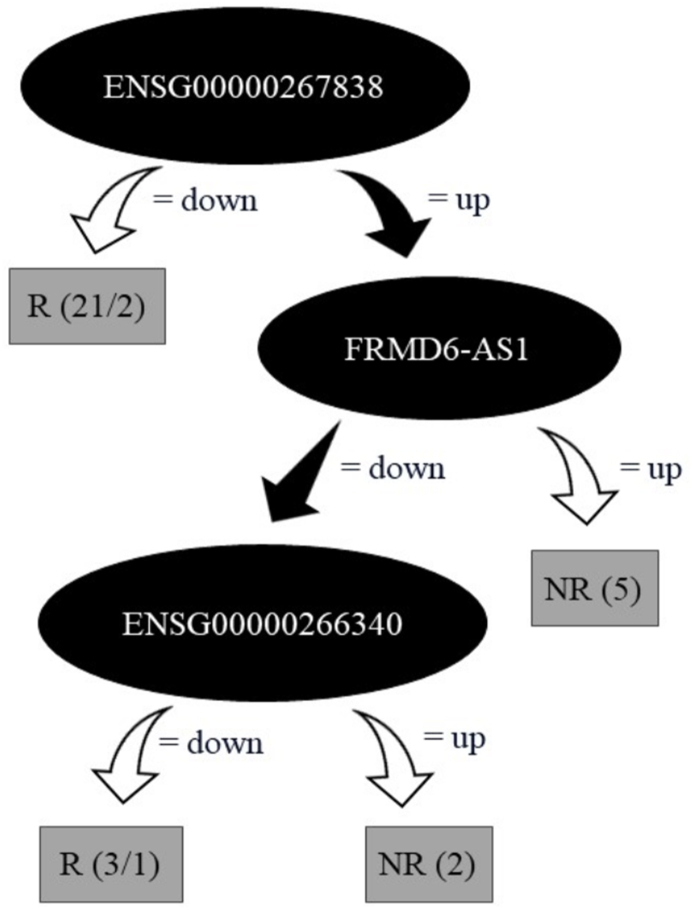
**– Decision tree generated from the 49 DEGs according to the lncRNAs gene expression profile for the 31 CC patients.** Using the full data set (Full training) to classify the records, the algorithm found 28/31 (90.32 %) possibilities. While the LOOCV validation method correctly classified 25/31 hits, i.e. 80.64 % accuracy.

Decision tree analysis was utilized to discern transcripts that could serve as biomarkers crucial in determining therapy responses, based on their expression profiles of either positive or negative regulation (upregulation and downregulation) for the R and NR patient groups. Among the 49 DEGs identified with CC expression associated, this analysis selected a highly informative subset of three lncRNAs: *ENSG00000267838*, *FRMD6-AS1*, and *ENSG00000266340*. The accuracy of the analysis was 90.32 % for FULL dataset training, and 80.64 % in LOOCV (Fig. 4).

**Fig. 5:**
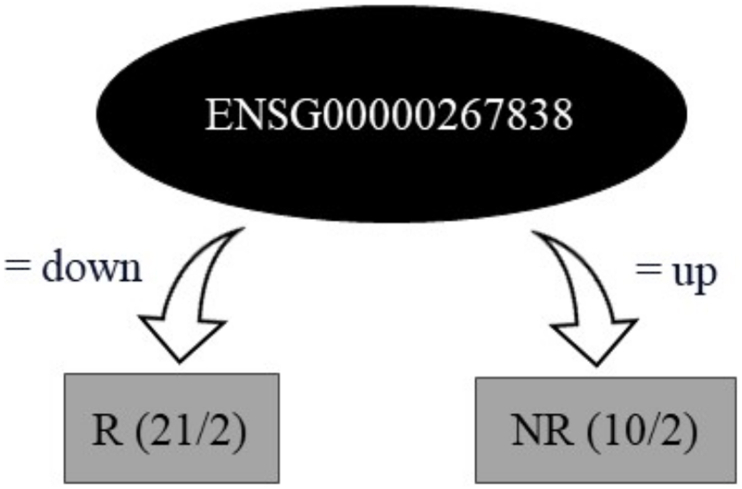
**– Decision tree generated for lncRNA *ENSG00000267838*.** An additional decision tree was built based on lncRNA ENSG00000267838 expressions to classify patients into groups of responders (R) and non-responders (NR) to treatment. The model obtained an accuracy of 80.64 % in the leave-one-out cross-validation (LOOCV) and an accuracy of 87.09 % in the full set analysis (FULL).

The analysis indicated that downregulation of *ENSG00000267838* and *ENSG0*0000266340 might be associated with a positive treatment response, while upregulation of these transcripts, along with *FRMD6-AS1*, could suggest a non-responsive behavior to therapy. A further decision tree focusing on *ENSG00000267838* reinforced the association of its downregulation with treatment responsiveness. In contrast, its upregulation was linked to non-responsiveness, achieving classification accuracies of 80.64 % (LOOCV) and 87.09 % (FULL), as illustrated in Fig. 5.

### 3.7 Sensitivity and specificity analysis

Sensitivity and specificity analysis was conducted to gauge the performance metrics of the model, aimed at delineating its capability to discern varying aspects of treatment response accurately. This method facilitated a deeper exploration of molecular determinants, such as the expression of DEGs, in relation to the efficacy of chemoradiotherapy in CC. The gene *ENSG00000267838*, as highlighted earlier, showcased a distinct capacity to categorize the 31 patients into responders (R, 21) and non-responders (NR, 10), predicated upon its expression being either positive or negative. This discriminatory power was further corroborated through sensitivity and specificity analysis, leveraging the expression profiles of the 11 DEGs across the 21 R and 10 NR patients. Considering the full training dataset, the model's overall accuracy was 87.09 %, with the LOOCV accuracy at 77.41 %. Sensitivity, or the model's proficiency in correctly identifying true positives, stood at 90.48 %, whereas specificity, indicating the accuracy in pinpointing true negatives, was 80 %. These metrics demonstrate the model's robustness, evidenced by high counts of True Positives (19) and True Negatives (8), alongside lower incidences of False Positives (2) and False Negatives (2), suggesting commendable classification performance. Moreover, the Positive Likelihood Ratio (4.52) and Negative Likelihood Ratio (0.12) further affirm the model's overall efficacy.

### 3.8 Progression-free survival analysis

**Fig. 6:**
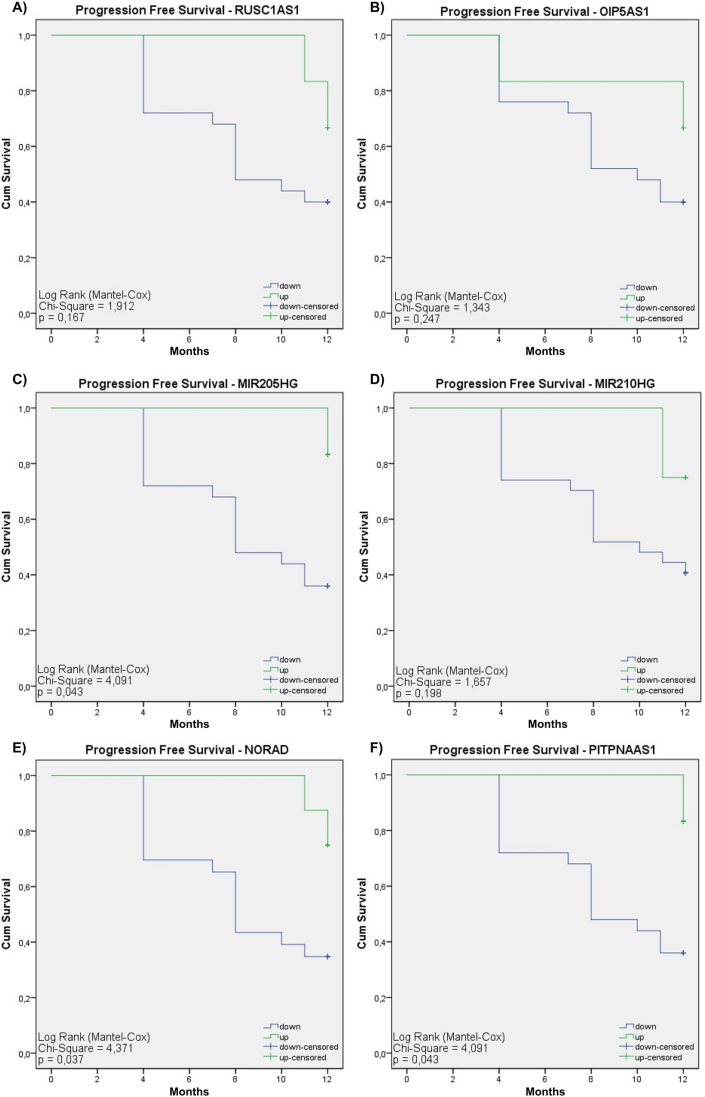

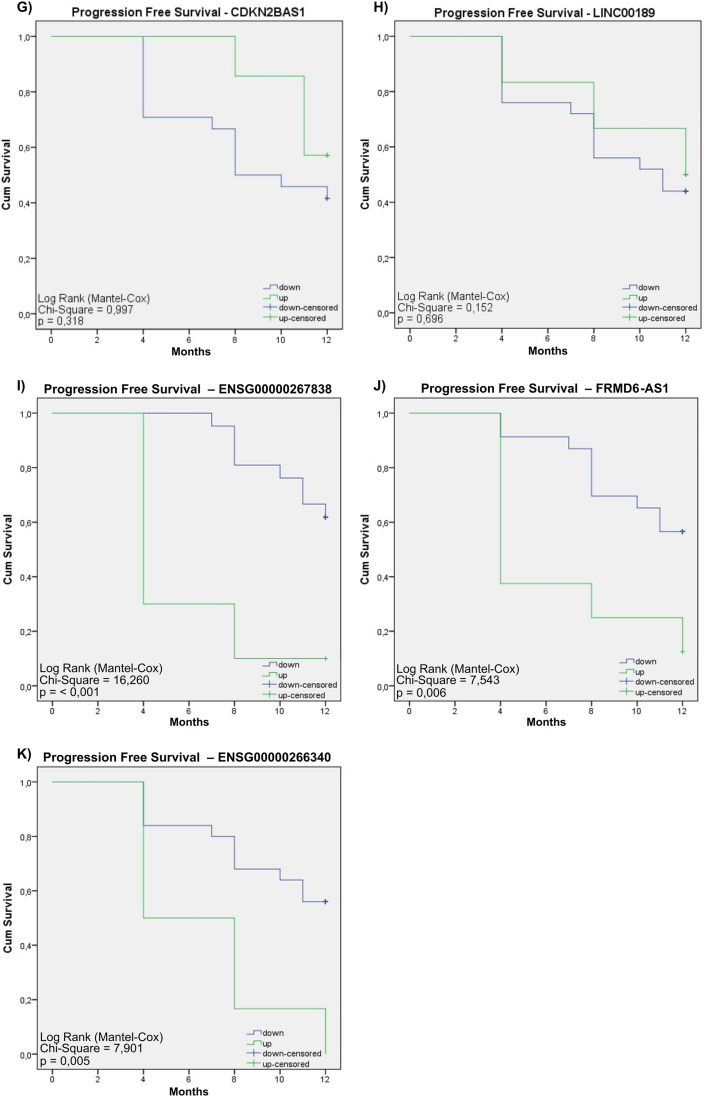
**– Kaplan-Meier graphs for the 11 transcripts that stood out as possible biomarkers for CC patients.** C, E, F, I, J, and K show a significant relationship with the prognosis of CC patients. All lncRNAs downregulated were associated with poor prognosis. However, the last three transcripts (*ENSG00000267838*, *FRMD6-AS1*, and *ENSG00000266340*) showed a high risk when positively regulated.

The Kaplan-Meier method was conducted to ascertain the relationship between the expression of various lncRNAs and adverse outcomes such as recurrence or mortality in CC patients. This examination focused on eleven lncRNAs; eight of these lncRNAs resulted from refining the 49 lncRNAs, and three were obtained through decision tree analysis. The study revealed distinct patterns of association between these lncRNAs and the patient's prognosis. Specifically, *MIR205HG*, *NORAD*, and *PITPNA-AS1* demonstrated statistical significance (p-value <0.05), linking their lower expression levels to a poorer prognosis. Conversely, *ENSG00000267838*, *FRMD6-AS1*, and *ENSG00000266340*, also achieving statistical significance, were associated with a higher risk when overexpressed, implicating their upregulation in disease progression, and reinforcing their potential value as prognostic biomarkers. For the remaining lncRNAs, including *RUSC1-AS1*, *OIP5-AS1*, *MIR210HG*, *CDKN2B-AS1*, and LINC00189, no significant differences in expression profiles were noted (Fig. 6).

**Fig. 7:**
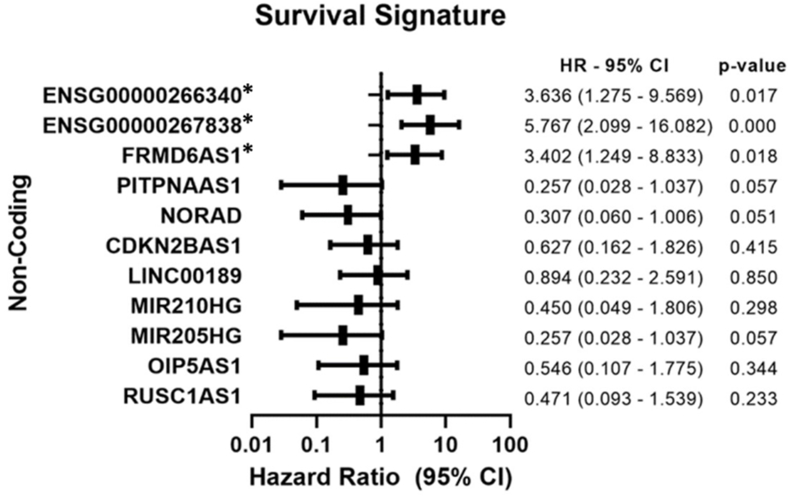
**– Cox proportional hazard model analysis.** This figure shows the evaluation of the hazard ratio for the lncRNAs identified, to determine their potential as prognostic biomarkers in CC. Cox analysis was used to quantify the relationship between lncRNA expression and patient progression-free survival. Hazard Ratio values greater than 1 indicate an increased risk of disease progression associated with high lncRNA expression, while values less than 1 suggest an association with a lower risk of progression.

The Cox proportional hazard model analysis was employed to compute hazard ratio values of the 11 lncRNAs under study and their respective confidence intervals (CIs). *ENSG00000267838*, *ENSG00000266340*, and *FRMD6-AS1,* when overexpressed, are significantly linked to an elevated risk of disease progression in CC patients. Notably, the lncRNA *ENSG00000267838* emerged as particularly significant, marked by a very low p-value (<0.001), thereby bolstering its importance as a prognostic biomarker for CC. Meanwhile, the under-expressed lncRNA *NORAD* was associated with a worse prognosis (p = 0.051) (Fig. 7).

Conversely, lncRNA LINC00189, despite suggesting a potentially favorable prognosis upon overexpression, did not achieve statistical significance, which could imply a non-systematic association. This observation aligns with the findings from the Kaplan-Meier survival analysis. Similar patterns were observed for *MIR210HG*, *CDKN2B-AS1*, *OIP5-AS1*, and *RUSC1-AS1*, indicating that the expression levels of these lncRNAs may play a critical role in predicting the prognosis of CC.

## 4 Discussion

CC exhibits significant global disparities, emphasizing the need for implementation studies to standardize care. This involves incorporating advancements like advanced imaging, radiotherapy, and biological products [21]. The WHO global strategy aims for a 90 % HPV vaccination rate, 70 % CC screening coverage, and 90 % treatment provision for precancerous lesions and invasive CC, anticipating a substantial reduction in CC incidence by 2100. Besides expanding therapy access, a deep understanding of tumor biology remains crucial [3]. State-of-the-art methods, such as RNA sequencing and artificial intelligence, understanding generate genomic findings, enabling gene expression analysis, biomarker identification, and understanding cancer progression [22].

In this context, we identified 417 lncRNAs with differential expression in the R and NR patient groups. The emergence of unique genetic signatures suggests the potential role of these lncRNAs as markers indicative of treatment response in CC. The reversal of gene expression patterns between the R and NR groups illustrates the complex molecular landscape driving the variability in treatment outcomes. Gene ontology analysis showed that the identified lncRNA genes, except for *SNHG29*, are involved in CC pathogenesis. These genes include *CDKN2B-AS1* [23], *NEAT1* [24], *MALAT1* [25], and *SCARNA9* [26]. The scientific literature has previously documented the roles of the latter four transcripts in CC, highlighting their potential significance in the disease's pathophysiology. These lncRNAs are linked to various facets of CC progression, accentuating their utility as molecular targets for diagnostic and therapeutic applications. The evaluation of these results suggests that although certain GO terms did not reach statistical significance, the documented involvement of these lncRNA genes in the scientific literature supports the critical function of lncRNAs in the context of CC.

Other studies have already carried out strategic filtering of DEGs by their expression in normal tissues and cancers of the female reproductive system [27,28], culminating in the identification of 49 transcripts considered potentially significant for the biological processes underlying CC. The genetic signature distinguishing the R and NR groups suggests that these DEGs may impact treatment outcomes or disease progression. This differentiation highlights the biological diversity in CC and emphasizes the necessity of adjusting therapeutic strategies to account for these variations.

In addition to exploring the mechanisms of gene regulation, our study highlights the relevance of functional interactions between lncRNAs and TFs in controlling chromatin remodeling processes. These interactions play a crucial role in modulating gene expression and directly affect key cellular processes such as senescence, metastasis, and resistance to conventional treatments. Our results demonstrate the complex network of interactions between lncRNAs, TFs, miRNAs, and proteins associated with differentially expressed genes, contributing to a deeper understanding of the biology of CC.

In investigating gene regulation mechanisms, our study delves into the role of long non-coding RNAs in modulating gene expression via interactions with TFs and their capacity to function as TFs themselves, thereby controlling chromatin remodeling processes [29]. Our results have demonstrated the impact of lncRNA interactions with biomolecules on key cellular processes, including senescence, metastasis, and resistance to radiation and chemotherapy, as detailed in Supplementary Table 2 [30]. Our research identified proteins linked to differentially expressed genes that play a crucial role in the complexity of cervical cancer, as detailed in Supplementary Table 3. Identifying a high mutational burden within regulatory proteins contributes to the emergence of factors leading to genomic instability in CC [31].

The TFs associated with lncRNAs of interest in the context of CC fulfill a variety of functions ranging from tumor suppression to the facilitation of tumor progression. These activities are pivotal in influencing overall and progression-free survival rates, advanced stages as per the FIGO classification, and the likelihood of cancer recurrence [32]. Functioning as RNA-binding proteins, these proteins regulate genetic elements, influencing transcripts, pathways, and processes such as ferroptosis [33]. Moreover, it should be noted that these proteins play a significant role in cell cycle progression, where they act as coactivators of TP53 or are critical in the TP53 degradation mediated by the HPV-16 viral oncoprotein E6. Such interactions are instrumental in altering miRNA profiles that affect cell motility, thereby influencing migration and invasion events [34]. Remarkably, these proteins are related to the progression of tumor size and other clinicopathological features, playing a key role in the dynamics of tumor progression with consequential effects on survival and clinical outcomes [35].

The miRNAs identified through the AnnoLnc2 tool are instrumental in the regulation of tumor growth and progression, employing complex mechanisms. This includes the interaction with lncRNAs, such as the lncRNA *MIR205HG*, which functions as a competing endogenous RNA and influences tumor proliferation by sequestering *miR-122-5p* in CC [36]. Furthermore, these miRNAs can modulate radiosensitivity and chemosensitivity, prompting cell cycle arrest and early apoptosis and serving as tumor suppressors [37]. In addition, our results highlight the importance of survival curve observations in assessing the impact of miRNAs on disease progression and overall survival in patients with CC. The survival analysis revealed significant connections between the expression of these miRNAs and overall survival, tumorigenesis, and migration in CC. They act as tumor suppressors and increase sensitivity to cisplatin, highlighting their significant potential as therapeutic targets and prognostic markers.

Refining the analysis to focus on interactions between transcripts and biomolecules led to identifying 8 DEGs that exhibit potential interactions and have specific regulatory patterns. These DEGs include *MIR205HG*, *NORAD*, LINC00189, *RUSC1-AS1*, *PITPNA-AS1*, *CDKN2B-AS1*, *MIR210HG*, and *OIP5-AS1*. This approach is useful for analyzing and identifying the most suitable candidates for future research when considering the involvement of lncRNAs [38]. The scientific literature and resources such as the UCSC Genome Browser have substantiated the associations of these lncRNAs with CC, reinforcing their potential roles and the need for further investigations in this context.

The decision tree analysis elucidated the connection between the expression profiles of selected DEGs, namely *ENSG00000267838*, also known as lnc-LENG9-5, *ENSG00000266340*, and *FRMD6-AS1*, and treatment response. Notably, *ENSG00000267838* demonstrated differential expression in female reproductive tissues, with a marked decrease in expression observed in CC, underscoring its significance within this context. The performance metrics of the model, with a sensitivity of 90.48 % and specificity of 80 %, demonstrate its effectiveness in correctly classifying patients into R and NR groups. The high rates of true positives and true negatives, coupled with low rates of false positives and false negatives, highlight the model's reliability. Its interaction with microRNAs, for example, miR-10-5p, implies an impact on cell viability, emphasizing its importance in CC [39]. Further decision tree analysis focusing exclusively on *ENSG00000267838* assessed this lncRNA's expression in R and NR groups. This detailed examination confirmed *ENSG00000267838*'s potential as a discriminative marker between R and NR groups. This suggests that its downregulation could indicate a positive response to treatment, whereas upregulation might signify treatment resistance.

During our comprehensive investigation of lncRNAs associated with CC, we identified several lncRNAs that play key roles in inhibiting apoptosis, promoting metastasis, contributing to FIGO stage progression, and that can act as competing endogenous RNAs (ceRNAs). One of these lncRNAs, *MIR205HG*, is implicated in CC progression through its function as a pro-tumorigenic ceRNA. It interacts with *SRSF1*, *KRT17*, *miR-16-5p*, and *FOXP2*, presenting a potential avenue for CC diagnosis and treatment [40,41]. *NORAD*, which is linked to a poor prognosis, functions as a ceRNA to promote cell proliferation and invasion by binding to *miR-590-3p* [42]. LINC00189 is notable for its predictive value regarding CC recurrence [43], while *RUSC1-AS1* serves as a ceRNA regulating *BCL-2* protein expression post-transcriptionally [44]. *PITPNA-AS1* influences proliferative, apoptotic, and cell cycle dynamics by interacting with miR-876-5p, underscoring its potential as a biomarker [45]. *CDKN2B-AS1* plays a role in CC progression through biological process regulation and acts as a ceRNA by binding to *miR-181a-5p* [46]. *MIR210HG* promotes CC progression by acting as a ceRNA and regulating the *miR-503-5p/TRAF4* axis [47]. The suppression of *OIP5-AS1* has been shown to reduce cell viability, invasion, and migration, impacting cell cycle regulation and apoptosis [48].

The identification of *ENSG00000267838*, *ENSG00000266340*, and *FRMD6-AS1* as key elements in distinguishing between groups of patients, as highlighted by the decision tree analysis, emphasizes the need to deepen the understanding of their specific roles in influencing responses to chemoradiotherapy. Regarding lncRNA *ENSG00000267838*, it exhibited positive results in response to chemoradiotherapy treatment, but a worse progression-free survival rate for the patient group. Exploring these mechanisms in greater depth is fundamental to developing more effective therapeutic strategies. Therefore, further studies are necessary to validate these findings, improve our understanding of the dynamics and influence of these DEGs in cervical cancer, and establish a more accurate signature for treatment response.

## 5 Conclusion

In summary, despite ongoing efforts to screen for CC and investigate biomarkers, there remains a pressing need for further research and exploring new avenues. This study underscores the pivotal role of lncRNAs in influencing therapeutic responses via their expression profiles, thereby reinforcing their potential utility as biomarkers for the diagnosis, prognosis, and monitoring of CC. Our findings particularly spotlight three lncRNAs—*ENSG00000267838*, *FRMD6-AS1*, and *ENSG00000266340*—as crucial in differentiating patient responses to therapy based on their regulatory profiles. The under-expression of *MIR205HG*, *NORAD*, and *PITPNA-AS1* was linked to a less favorable prognosis, whereas the over-expression of *ENSG00000267838*, *FRMD6-AS1*, and *ENSG00000266340* was indicative of a higher risk of disease progression. Among these, lncRNA *ENSG00000267838* emerges as a standout biomarker, exhibiting a significant capability to segregate responders from non-responders, highlighting its potential in guiding therapeutic decisions and patient management in CC.

## CRediT authorship contribution statement

**Bruna Custódio Dias Duarte:** Writing – review & editing, Writing – original draft, Visualization, Validation, Supervision, Software, Project administration, Methodology, Investigation, Data curation, Conceptualization. **Fábio Ribeiro Queiroz:** Writing – review & editing, Writing – original draft, Visualization, Validation, Supervision, Software, Project administration, Methodology, Investigation, Data curation, Conceptualization. **Álvaro Percínio Costa:** Writing – review & editing, Visualization, Validation, Data curation. **Angelo Borges de Melo Neto:** Writing – review & editing, Software, Investigation, Formal analysis, Data curation. **Carolina Pereira de Souza Melo:** Writing – review & editing, Validation, Methodology, Investigation, Data curation. **Paulo Guilherme de Oliveira Salles:** Writing – review & editing, Validation, Methodology, Investigation. **Wander de Jesus Jeremias:** Writing – review & editing, Visualization, Supervision, Project administration. **Pedro Luiz Lima Bertarini:** Writing – review & editing, Software, Formal analysis, Data curation. **Laurence Rodrigues do Amaral:** Writing – review & editing, Software, Formal analysis, Data curation. **Letícia da Conceição Braga:** Writing – review & editing, Visualization, Supervision, Resources, Project administration, Funding acquisition. **Matheus de Souza Gomes:** Writing – review & editing, Visualization, Supervision, Resources, Project administration, Funding acquisition. **Agnaldo Lopes da Silva Filho:** Writing – review & editing, Supervision, Project administration, Funding acquisition.

## Funding

This work was supported by the Brazilian Ministry of Health for grants (Programa Nacional de Apoio à Atenção Oncológica - Pronon: NUP:25000.159953/2014–18 and NUP:25000.079266/2015–09); and the Fundação de Amparo à Pesquisa do Estado de Minas Gerais – FAPEMIG: APQ-02255-22, Belo Horizonte, MG, Brazil. Rede Mineira de Pesquisa Transactional em Oncologia – RMPTO: FAPEMIG - RED00059-23.

## Declaration of competing interest

The authors declare that they have no known competing financial interests or personal relationships that could have appeared to influence the work reported in this paper.
