# Supplementary material for: Upregulation of long non-coding RNA ENSG00000267838 is related to the high risk of progression and non-response to chemoradiotherapy treatment for cervical cancer
Source: Noncoding RNA Res. 2024 Oct 24;11:104–14. doi: 10.1016/j.ncrna.2024.10.004 (PMC11683307; doi:10.1016/j.ncrna.2024.10.004)
Supplement: Multimedia component 1 [file mmc1.docx]

**Supplementary Table 1 - 49 DEGs selected by AnnoLnc2.** ENSEMBL ID, symbol and description of the 49 DEGs selected for their involvement in tissues and cancers of the female reproductive system.

| ENSEMBL ID | **SYMBOL** | **DESCRIPTION** |
| --- | --- | --- |
| ENSG00000215533 | *LINC00189* | Long Intergenic Non-Protein  Coding RNA 189 |
| ENSG00000225855 | *RUSC1-AS1* | RUSC1 Antisense RNA 1 |
| ENSG00000230937 | *MIR205HG* | MIR205 host gene |
| ENSG00000236618 | *PITPNA-AS1* | PITPNA Antisense RNA 1 |
| ENSG00000238142 | LOC105376805 | Uncharacterized LOC105376805 |
| ENSG00000240498 | *CDKN2B-AS1* | CDKN2B Antisense RNA 1 |
| ENSG00000244151 | RP11-148K1.12 | Novel transcript |
| ENSG00000247095 | *MIR210HG* | MIR210 Host Gene |
| ENSG00000247556 | *OIP5-AS1* | OIP5 Antisense RNA 1 |
| ENSG00000248980 | SPCS3-AS1 | SPCS3 Antisense RNA 1 |
| ENSG00000251562 | MALAT1 | Metastasis Associated Lung Adenocarcinoma Transcript 1 |
| ENSG00000257379 | RP11-793H13.8 | Novel transcript |
| ENSG00000257553 | RP11-603J24.17 | Novel transcript |
| ENSG00000257663 | RP11-1100L3.7 | Novel transcript |
| ENSG00000258017 | RP11-386G11.10 | Novel transcript |
| ENSG00000258232 | RP11-161H23.5 | Novel transcript |
| ENSG00000259865 | RP11-488L18.10 | Novel transcript |
| ENSG00000259972 | AC009120.6 | Novel transcript |
| ENSG00000260032 | *NORAD* | Non-Coding RNA Activated By DNA Damage |
| ENSG00000261061 | RP11-303E16.2 | Novel transcript |
| ENSG00000261183 | SPINT1-AS1 | SPINT1 Antisense RNA 1 |
| ENSG00000264577 | AC010761.8 | Novel transcript |
| ENSG00000265401 | RP11-138I1.4 | Novel transcript |
| *ENSG00000266340* | RP11-848P1.4 | Novel transcript |
| ENSG00000266402 | SNHG25 | Small Nucleolar RNA Host Gene 25 |
| ENSG00000267165 | CHMP1B-AS1 | CHMP1B Antisense RNA 1 |
| ENSG00000267523 | CTD-2537I9.12 | Novel transcript |
| ENSG00000267815 | CTB-191K22.5 | Novel transcript |
| *ENSG00000267838* | AC008746.12 | Novel transcript |
| ENSG00000269243 | CTD-2231E14.8 | Novel transcript |
| ENSG00000269680 | CTD-3128G10.6 | Novel transcript |
| ENSG00000269893 | SNHG8 | Small Nucleolar RNA Host Gene 8 |
| ENSG00000269958 | RP11-73M18.8 | Novel transcript |
| ENSG00000272182 | RP11-802O23.3 | Novel transcript |
| ENSG00000272234 | CTD-2325A15.5 | Novel transcript |
| ENSG00000272288 | ILRUN-AS1 | Novel transcript |
| ENSG00000272696 | RP11-339B21.13 | Novel transcript |
| ENSG00000272933 | TRIM8-DT | TRIM8 Divergent Transcript |
| ENSG00000273149 | RP11-290D2.6 | Novel transcript |
| ENSG00000273449 | RP11-218F10.3 | Novel transcript |
| ENSG00000273888 | *FRMD6-AS1* | FRMD6 Antisense RNA 1 |
| ENSG00000276570 | CTD-2587H24.14 | Novel transcript |
| ENSG00000277801 | RP11-681H18.2 | Novel transcript |
| ENSG00000277978 | RP11-403P17.6 | Novel transcript |
| ENSG00000279364 | RP11-463I20.1 | TEC (To be Experimentally Confirmed) |
| ENSG00000279605 | - | TEC (To be Experimentally Confirmed) |
| ENSG00000279753 | - | TEC (To be Experimentally Confirmed) |
| ENSG00000280027 | - | Novel transcript |
| ENSG00000280064 | - | Novel transcript |
