# Supplementary material for: Upregulation of long non-coding RNA ENSG00000267838 is related to the high risk of progression and non-response to chemoradiotherapy treatment for cervical cancer
Source: Noncoding RNA Res. 2024 Oct 24;11:104–14. doi: 10.1016/j.ncrna.2024.10.004 (PMC11683307; doi:10.1016/j.ncrna.2024.10.004)
Supplement: Multimedia component 2 [file mmc2.docx]

**Supplementary Table 2** **- Transcription factors that are associated with the 49 DEGs.** Symbol of the transcription factors, their name, their function in cervical cancer and their interactions with the 49 selected lncRNAs, located by AnnoLnc2.

| TFs | Name | Function in the CC | LncRNAs of interaction |
| --- | --- | --- | --- |
| AMH | Anti-Müllerian Hormone | Tumor supressor.  [[1]](https://link.springer.com/article/10.1007/s10815-019-01622-7) | LINC00189, RUSC1-AS1, ENSG00000238142, CDKN2B-AS1, MIR210HG, SPCS3-AS1, MALAT1, ENSG00000257379, ENSG00000257553, ENSG00000257663, ENSG00000258232, ENSG00000265401, SNHG25, ENSG00000272182, ENSG00000272234, ENSG00000273449, ENSG00000277801 |
| ASF1A | Anti-Silencing Function 1A Histone Chaperone | Correlation with increased rates of cancer progression and occurrence of metastases.  Contributes to DNA repair and cellular senescence.  [[2]](https://www.nature.com/articles/s41419-020-02872-5) | LINC00189, RUSC1-AS1, CDKN2B-AS1, SPCS3-AS1, MALAT1, ENSG00000257379, ENSG00000257553, ENSG00000257663, ENSG00000258232, CHMP1B-AS1, SNHG8, TRIM8-DT |
| BRCA1 | Breast Cancer 1 | Expression related to survival, low expression is associated with a worse prognosis.  [[3]](https://www.ncbi.nlm.nih.gov/pmc/articles/PMC8606581/) | LINC00189, PITPNA-AS1, CDKN2B-AS1, ENSG00000244151, OIP5-AS1, MALAT1, ENSG00000257379, ENSG00000257553, ENSG00000257663, ENSG00000258232, ENSG00000273888, NORAD, ENSG00000265401, SNHG25, CHMP1B-AS1, SNHG8, ILRUN-AS1, TRIM8-DT, ENSG00000273149, ENSG00000279605 |
| BRD4 | Bromodomain Containing 4 | Repression leads to significant radiosensitization and potentiation of DNA damage.  [[4]](https://www.nature.com/articles/s41388-021-01735-3) | LINC00189, RUSC1-AS1, ENSG00000238142, ENSG00000244151, MIR210HG, OIP5-AS1, SPCS3-AS1, MALAT1, ENSG00000257379, ENSG00000257553, ENSG00000257663, ENSG00000258232, NORAD, NORAD, ENSG00000265401, CHMP1B-AS1, SNHG8, TRIM8-DT, ENSG00000273149, ENSG00000279605 |
| CEBPB  (C/EBPβ) | CCAAT Enhancer Binding Protein Beta | Overexpression can inhibit cell proliferation, migration, invasion, arrest cells in S phase and promote apoptosis.  [[5]](https://www.ncbi.nlm.nih.gov/pmc/articles/PMC9872280/) | LINC00189, RUSC1-AS1, PITPNA-AS1, CDKN2B-AS1, ENSG00000244151, OIP5-AS1, MALAT1, ENSG00000257379, ENSG00000257553, ENSG00000257663, ENSG00000258017, ENSG00000258232, SPINT1-AS1, ENSG00000264577, SNHG25, CHMP1B-AS1, ENSG00000267523, ENSG00000267815, ENSG00000269243, ENSG00000269680, SNHG8, ILRUN-AS1, TRIM8-DT, ENSG00000273149, ENSG00000279364, ENSG00000279605 |
| CTCF | CCCTC-Binding Factor | Integrated into HPV, it reorganizes the chromatin state and positively regulates genes essential for tumor viability.  [[6]](https://genomebiology.biomedcentral.com/articles/10.1186/s13059-023-02926-9) | LINC00189, RUSC1-AS1, PITPNA-AS1, ENSG00000238142, CDKN2B-AS1, ENSG00000244151, MIR210HG, OIP5-AS1, SPCS3-AS1, MALAT1, ENSG00000257379, ENSG00000257553, ENSG00000257663, ENSG00000258232, ENSG00000273888, ENSG00000259972, NORAD, ENSG00000265401, SNHG25, CHMP1B-AS1, ENSG00000267523, ENSG00000267815, ENSG00000267838, ENSG00000269243, SNHG8, ENSG00000272234, TRIM8-DT, ENSG00000273149, ENSG00000279364, ENSG00000279605, ENSG00000279753 |
| DEK | DEK proto-oncogene | Overexpression inhibits apoptosis and senescence in HeLa cells, and prevents tp53 transcriptional activity.  [[7]](https://link.springer.com/article/10.1007/s10147-020-01735-5) | LINC00189, RUSC1-AS1, PITPNA-AS1, CDKN2B-AS1, MALAT1, ENSG00000257379, ENSG00000257553, ENSG00000258232, ENSG00000259972, NORAD, ENSG00000265401, SNHG25, CHMP1B-AS1, ENSG00000267523, TRIM8-DT, ENSG00000279605. |
| E2F1 | E2F transcription factor 1 | Prognostic factor, favorable when overexpressed. Up-regulation promotes apoptosis through tp53-dependent and -independent pathways.  [[8]](https://doi.org/10.1016/j.yexmp.2020.104368) | RUSC1-AS1, PITPNA-AS1, ENSG00000238142, CDKN2B-AS1, ENSG00000244151, MIR210HG, OIP5-AS1, SPCS3-AS1, MALAT1, ENSG00000257379, ENSG00000257553, ENSG00000257663, ENSG00000258017, ENSG00000258232, ENSG00000273888, NORAD, ENSG00000265401, SNHG25, CHMP1B-AS1, ENSG00000267523, SNHG8, ILRUN-AS1, ENSG00000272696, TRIM8-DT, ENSG00000273149, ENSG00000279605 |
| ELK1 | ETS Transcription Factor ELK1 | Increased expression represses apoptosis and autophagy in CC cells.  [[9]](https://www.spandidos-publications.com/ol/13/5/2949) [[10]](https://www.ncbi.nlm.nih.gov/pmc/articles/PMC6914414/) | LINC00189, PITPNA-AS1, CDKN2B-AS1, MALAT1, ENSG00000257379, ENSG00000257553, ENSG00000258232, NORAD, ENSG00000265401, ENSG00000273149 |
| ELK4 | ETS Transcription Factor ELK4 | Promotes cell cycle progression.  [[11]](https://balkanmedicaljournal.org/uploads/pdf/pdf_BMJ_2521.pdf) | LINC00189, RUSC1-AS1, PITPNA-AS1, CDKN2B-AS1, MALAT1, ENSG00000257379, ENSG00000257553, ENSG00000258017, ENSG00000258232, NORAD, ENSG00000265401, SNHG25, CHMP1B-AS1, ENSG00000272234, TRIM8-DT, ENSG00000273149 |
| EP300 | E1A Binding Protein P300 | Inhibitor of the anti-tumor immune response in CC via metabolic modulation.  [[12]](https://www.spandidos-publications.com/10.3892/mmr.2021.12388) [[13]](https://www.sciencedirect.com/science/article/pii/S2405844023021035) | LINC00189, ENSG00000264577, RUSC1-AS1, PITPNA-AS1, CDKN2B-AS1, ENSG00000244151, OIP5-AS1, SPCS3-AS1, MALAT1, ENSG00000257379, ENSG00000257553, ENSG00000257663, ENSG00000258232, ENSG00000273888, ENSG00000259972, NORAD, ENSG00000265401, CHMP1B-AS1, SNHG8, ILRUN-AS1, TRIM8-DT, ENSG00000273149 |
| FOXM1 | Forkhead Box M1 | Drug resistance and poor prognosis.  [[14]](https://www.ncbi.nlm.nih.gov/pmc/articles/PMC8820921/) [[15]](https://www.gynecologiconcology-online.net/article/S0090-8258(12)00729-9/fulltext) | LINC00189, RUSC1-AS1, PITPNA-AS1, ENSG00000244151, OIP5-AS1, SPCS3-AS1, MALAT1, ENSG00000257379, ENSG00000257553, ENSG00000257663, ENSG00000258017, ENSG00000258232, NORAD, SNHG25, CHMP1B-AS1, SNHG8, ENSG00000273149 |
| JUND | JunD proto-oncogene | Increases cell proliferation and the ability of cancer cell lines to invade.  [[16]](https://www.ncbi.nlm.nih.gov/pmc/articles/PMC10239735/) | LINC00189, RUSC1-AS1, CDKN2B-AS1, OIP5-AS1, MALAT1, ENSG00000257379, ENSG00000258232, ENSG00000259972, NORAD, SPINT1-AS1, SNHG25, CHMP1B-AS1 |
| MCM7 | Minichromosome Maintenance Complex Component 7 | Associated with high-grade cervical lesions, tumor formation, progression and malignant conversion.  [[17]](https://www.sciencedirect.com/science/article/pii/S0046817716301654) [[18]](https://ijgc.bmj.com/content/23/2/318) [[19]](https://www.ncbi.nlm.nih.gov/pmc/articles/PMC3280060/) | LINC00189, RUSC1-AS1, ENSG00000238142, ENSG00000244151, MIR210HG, OIP5-AS1, SPCS3-AS1, SPCS3-AS1, MALAT1, ENSG00000257379, ENSG00000257553, ENSG00000257663, ENSG00000258232, NORAD, ENSG00000265401, SNHG25, ENSG00000267523, SNHG8, ENSG00000272234, ENSG00000279605, ENSG00000279753 |
| MYBL2 | MYB Proto-Oncogene Like 2 | Expression levels affected by HPV 16. Expression levels are related to pathological changes in the cervix and may induce the development of CC.  [[20]](https://link.springer.com/protocol/10.1007/978-1-4939-2013-6_18) [[21]](https://www.frontiersin.org/journals/oncology/articles/10.3389/fonc.2022.816070/full) [[22]](https://pesquisa.bvsalud.org/portal/resource/pt/wpr-606627) | LINC00189, CDKN2B-AS1, MALAT1 |
| MYC | MYC proto-oncogene | Simultaneous overexpression of HPV amplification, which is associated with the acquisition of a malignant phenotype in cervical cells. It is an oncogene involved in tumor progression.  [[23]](https://www.spandidos-publications.com/10.3892/ol.2018.9825) [[24]](https://www.sciencedirect.com/science/article/pii/S0090825814007975) | LINC00189, RUSC1-AS1, PITPNA-AS1, CDKN2B-AS1, ENSG00000244151, MIR210HG, OIP5-AS1, SPCS3-AS1, MALAT1, ENSG00000257379, ENSG00000257553, ENSG00000257663, ENSG00000258017, ENSG00000258232, ENSG00000273888, NORAD, SPINT1-AS1, ENSG00000265401, SNHG25, CHMP1B-AS1, ENSG00000269243, SNHG8, ILRUN-AS1, TRIM8-DT, ENSG00000273149, ENSG00000279605 |
| NFE2L2 | NFE2 Like BZIP Transcription Factor 2 | It is associated with a poor prognosis and can affect the tumor microenvironment and macrophage infiltration.  [[25]](https://www.sciencedirect.com/science/article/pii/S1567576922007573) | LINC00189, RUSC1-AS1, PITPNA-AS1, CDKN2B-AS1, OIP5-AS1, MALAT1, ENSG00000257379, ENSG00000257553, ENSG00000258232, NORAD, SNHG25, CHMP1B-AS1, SNHG8, TRIM8-DT, ENSG00000273149 |
| NR2C2 | Nuclear receptor subfamily 2, group C, member 2 | Related to apoptosis mediated by DNA damage induced by ionizing radiation.  [[26]](https://doi.org/10.1016/j.bbrc.2018.11.110) | LINC00189, RUSC1-AS1, PITPNA-AS1, ENSG00000257379, ENSG00000257553, ENSG00000257663, ENSG00000258232, NORAD |
| ORC1 | Origin Recognition Complex subunit 1 | Overexpression accelerates the progression of CC, promoting proliferation and reducing apoptosis.  [[27]](https://www.tandfonline.com/doi/full/10.1080/21655979.2022.2078562) | RUSC1-AS1, MIR210HG, SPCS3-AS1, MALAT1, ENSG00000257553, ENSG00000257663, ENSG00000273888, NORAD, CHMP1B-AS1, ENSG00000267523 |
| PTEN | Phosphatase and Tensin Homolog | Under-expression is involved in pathogenesis, invasion and metastasis.  [[28]](https://doi.org/10.3892/br.2014.298) | LINC00189, MIR205HG, PITPNA-AS1, ENSG00000238142, CDKN2B-AS1, ENSG00000244151, MIR210HG, OIP5-AS1, SPCS3-AS1, MALAT1, ENSG00000257379, ENSG00000257553, ENSG00000257663, ENSG00000259972, NORAD, SPINT1-AS1, ENSG00000265401, CHMP1B-AS1, ENSG00000269243, TRIM8-DT |
| PYGO2 | Pygopus Family PHD Finger 2 | Essential for the growth of CC cell lines.  [[29]](https://www.sciencedirect.com/science/article/pii/S2211124718305497) | LINC00189, MALAT1, ENSG00000257379, ENSG00000257553,  ENSG00000257663, NORAD, SNHG25 |
| RAD21 | RAD21 Cohesin Complex Component | Associated with survival and susceptibility to CC.  [[30]](https://www.ncbi.nlm.nih.gov/pmc/articles/PMC6051231/) | LINC00189, RUSC1-AS1, PITPNA-AS1, CDKN2B-AS1, ENSG00000244151, MIR210HG, OIP5-AS1, SPCS3-AS1, MALAT1, ENSG00000257379, ENSG00000257553, ENSG00000257663, ENSG00000258232, ENSG00000273888, ENSG00000259972, NORAD, SPINT1-AS1, ENSG00000265401, SNHG25, CHMP1B-AS1, ENSG00000267523, ENSG00000267815, ENSG00000267838, ENSG00000269243, SNHG8, ILRUN-AS1, TRIM8-DT, ENSG00000273149, ENSG00000279364, ENSG00000279605 |
| SFMBT1 | Scm like with Four MBT domains 1 | Overexpression induced EMT and increased migration and invasion.  [[31]](https://www.ncbi.nlm.nih.gov/pmc/articles/PMC5216747/) | LINC00189, RUSC1-AS1, MALAT1, SNHG25, ENSG00000280064 |
| STAT1 | Signal Transducer and Activator of Transcription 1 | Associated with the progression of cervical lesions. Its expression can be affected by the viral load of HPV16. Increases the sensitivity of cervical tumor cells to chemotherapy drugs and radiation.  [[32]](https://www.spandidos-publications.com/10.3892/ol.2020.11889) [[33]](https://www.mdpi.com/2073-4425/14/6/1141) | LINC00189, RUSC1-AS1, PITPNA-AS1, CDKN2B-AS1, ENSG00000244151, MIR210HG, OIP5-AS1, SPCS3-AS1, MALAT1, ENSG00000257379, ENSG00000257553, ENSG00000257663, ENSG00000258232, ENSG00000259972, NORAD, SPINT1-AS1, SNHG25, CHMP1B-AS1, SNHG8, ILRUN-AS1, ENSG00000273149 |
| STAT3 | Signal Transducer and Activator of Transcription 3 | Promotes proliferation and migration skills.  [[34]](https://www.ncbi.nlm.nih.gov/pmc/articles/PMC8772194/) | LINC00189, CDKN2B-AS1, MALAT1, ENSG00000257379 |
| TFAP2A | Transcription Factor AP-2 Alpha | Associated with a higher tumor stage, lymph node metastasis and poor survival.  [[35]](https://www.ncbi.nlm.nih.gov/pmc/articles/PMC10170487/) | LINC00189, RUSC1-AS1, CDKN2B-AS1, MIR210HG, MALAT1, ENSG00000257379, ENSG00000257553, ENSG00000257663, ENSG00000258232, ENSG00000273888 |
| YY1 | YY1 transcription fator | It acts on the progression of HPV-positive CC by regulating the expression of viral oncogenes E6 and E7. It is involved in the tumorigenesis process. When inhibited, it induces tp53 activation and apoptosis in HPV-infected HeLa cells.  [[36]](https://www.ncbi.nlm.nih.gov/pmc/articles/PMC8998550/) | RUSC1-AS1, PITPNA-AS1, ENSG00000244151, OIP5-AS1, MALAT1, ENSG00000257379, ENSG00000258017, ENSG00000258232, NORAD, SNHG25, SNHG8, ENSG00000273149 |
