# Supplementary material for: Upregulation of long non-coding RNA ENSG00000267838 is related to the high risk of progression and non-response to chemoradiotherapy treatment for cervical cancer
Source: Noncoding RNA Res. 2024 Oct 24;11:104–14. doi: 10.1016/j.ncrna.2024.10.004 (PMC11683307; doi:10.1016/j.ncrna.2024.10.004)
Supplement: Multimedia component 3 [file mmc3.docx]

**Supplementary Table 3 - Proteins interacting with the 49 DEGs.** Symbol of proteins, their name, their function in CC and their interactions with the 49 selected lncRNAs localized by AnnoLnc2**.**

| Protein | Name | Function in the CC | LncRNAs of interaction |
| --- | --- | --- | --- |
| AEG-1/ MTDH | Astrocyte Elevated Gene-1/ Metadherin | Involved in EMT control. Induces chemoresistance. Up-regulation is correlated with tumor growth and lymph node metastasis. AEG-1 knockdown weakens the stem-like property of HeLa cells. AEG-1 staining is related to the increasing degree of the lesion in cervical intraepithelial neoplasia (CIN).  [[37]](https://pubmed.ncbi.nlm.nih.gov/24256614/) [[38]](https://www.ncbi.nlm.nih.gov/pmc/articles/PMC4111716/) [[39]](https://www.ncbi.nlm.nih.gov/pmc/articles/PMC3866971/) | SNHG8 |
| ALKBH5 | AlkB Homolog 5, RNA Demethylase | High expression is associated with a poor prognosis in patients and the induction of tumorigenesis and metastasis.  [[40]](https://www.sciencedirect.com/science/article/pii/S1043661823002190) | ENSG00000279605 |
| ARHGEF28 | Rho guanine nucleotide exchange factor 28 | It is prognostic for CC, high expression is favorable. Mutations influence the survival rate.  [[41]](https://www.proteinatlas.org/ENSG00000214944-ARHGEF28/pathology/cervical+cancer) | ENSG00000258017 |
| ASPH | Aspartate Beta-Hydroxylase | High expression is not favorable for the prognosis of CC. Studies show high expression in patients resistant to radiotherapy, as well as lower overall survival and progression-free survival.  [[42]](https://www.proteinatlas.org/ENSG00000198363-ASPH/pathology/cervical+cancer) [[43]](https://www.ncbi.nlm.nih.gov/pmc/articles/PMC9373049/) | ENSG00000279605 |
| ATXN2 | Ataxin 2 | Acts as a direct RNA-binding protein (RBP) in CC HeLa cells. It is involved in the regulation of global RNA processing and ribosomal translation.  [[44]](https://www.ncbi.nlm.nih.gov/pmc/articles/PMC5243904/) | ILRUN-AS1, ENSG00000277978, SNHG8, ENSG00000257663, RUSC1-AS1, ENSG00000266340, ENSG00000259972, ENSG00000259865, MALAT1, ENSG00000269680, MIR205HG, FRMD6-AS1, OIP5-AS1, ENSG00000276570, NORAD, ENSG00000279605 |
| CAPRIN1 | Cycle Associated Protein 1 | Involved in cell proliferation in Hela cells from CC.  [[45]](https://www.sciencedirect.com/science/article/pii/S0344033817302790) | ENSG00000279605, NORAD, SNHG8, ENSG00000269680, ENSG00000276570, ENSG00000269958 |
| CCAR1 | Cell Division Cycle And Apoptosis Regulator 1 | Expressed at all stages, suggesting a role in driving cell cycle progression. Acts as a coactivator of the tumor suppressor protein tp53.  [[46]](https://link.springer.com/article/10.1007/s13277-010-0151-4) | SPINT1-AS1, ENSG00000280064, ENSG00000266340, MIR205HG, MALAT1, OIP5-AS1, FRMD6-AS1, ENSG00000279364, ENSG00000269958, NORAD, ENSG00000279605, ENSG00000261061 |
| CIRBP | Cold Inducible RNA Binding Protein | It is prognostic of CC, high expression is favorable. Involved in cell metabolism and DNA methylation as prognostic molecular biomarkers in gynecological cancer.  [[47]](https://www.proteinatlas.org/ENSG00000099622-CIRBP) [[48]](https://www.ncbi.nlm.nih.gov/pmc/articles/PMC7814251/) | ENSG00000279605, NORAD |
| DDX3X | DEAD-box helicase 3 X-linked | It plays an oncogenic role in CC.  [[49]](https://www.mdpi.com/1422-0067/16/7/15578) [[50]](https://www.ncbi.nlm.nih.gov/pmc/articles/PMC6982152/) | ILRUN-AS1, SNHG8, ENSG00000279605, ENSG00000238142 |
| DGCR8 | DGCR8 Microprocessor Complex Subunit | Overexpressed in CC. Its expression is increased by HPV16 E7 to help accelerate cell proliferation and inhibit paclitaxel-induced cell apoptosis.  [[51]](https://doi.org/10.1016/j.cancergen.2020.09.003) [[52]](https://www.ncbi.nlm.nih.gov/pmc/articles/PMC4991410/) | ENSG00000279605, NORAD, TRIM8-DT |
| DROSHA | Drosha ribonuclease III | Increased expression in HPV-positive lineages by the viral oncoproteins E6 and E7 leads to miRNA alterations associated with greater cell motility in CC.  [[53]](https://www.sciencedirect.com/science/article/abs/pii/S2210776220302726) [[54]](https://www.ncbi.nlm.nih.gov/pmc/articles/PMC5499708/) | ENSG00000261061, ENSG00000267838, ENSG00000279753, MIR210HG, ENSG00000272696, ENSG00000259865, LINC00189, MIR205HG, OIP5-AS1, FRMD6-AS1, ENSG00000279364, ENSG00000276570, ENSG00000269958, ENSG00000279605, PITPNA-AS1 |
| EIF3A | Eukaryotic Translation Initiation Factor 3 Subunit A | High expression is associated with better survival and a better prognosis, as its expression is completely lost in CC after the cells reach a differentiated status.  [[55]](https://linkinghub.elsevier.com/retrieve/pii/S0304383517306274) | ENSG00000264577, ENSG00000257663, ENSG00000258017, MALAT1, ENSG00000269680, ENSG00000279605, ENSG00000273149 |
| EIF3D | Eukaryotic Translation Initiation Factor 3 Subunit D | Promotes tumor progression.  [[56]](https://www.tandfonline.com/doi/full/10.1080/01443615.2022.2130200) | ENSG00000273149, ENSG00000279364, ENSG00000279605, NORAD |
| ELAVL1 | ELAV like RNA binding protein 1 | Promotes the growth of cancer cells by regulating RNA in the cell cytoplasm.  [[57]](https://link.springer.com/article/10.1007/s11033-022-07868-2) | SNHG8 |
| EP300 | E1A Binding Protein P300 | Decreased expression in CC, reducing histone lactation modification and transcriptional mutation of cervical cancer cells, destabilizing cancer cell proliferation.  [[58]](https://www.sciencedirect.com/science/article/pii/S2405844023021035) | ENSG00000264577, ENSG00000257663, ENSG00000259972, ENSG00000259865, MALAT1, NORAD, FRMD6-AS1, ENSG00000269680, ENSG00000269958, ENSG00000279605 |
| EPPK1 | Epiplakin 1 | Expression positively correlated with tumor size in clinicopathological characteristics.  [[59]](https://link.springer.com/article/10.1186/s12885-021-08040-y) | SNHG25, ENSG00000257663, ENSG00000258017, ENSG00000259972, MALAT1, ENSG00000269958, ENSG00000279605 |
| HELLS | Helicase, Lymphoid Specific | HELLS is prognostic, high expression is favorable in CC. Promotes CC proliferation by regulating Nrf2-mediated ferroptosis.  [[60]](https://www.proteinatlas.org/ENSG00000119969-HELLS/pathology/cervical+cancer) [[61]](https://doi.org/10.21203/rs.3.rs-2689719/v1) | ENSG00000257553, ENSG00000273449, ENSG00000276570 |
| [HNRNPU](http://www.ncbi.nlm.nih.gov/gene?term=(HNRNPU%5BGene%2FProtein%20Name%5D)%20AND%20Homo%20sapiens%5BOrganism%5D) | heterogeneous nuclear ribonucleoprotein U | It has CC proliferative properties.  [[62]](https://www.nature.com/articles/s41419-022-05376-6) | MIR205HG, NORAD, ENSG00000269958, ENSG00000279605 |
| HUWE1 | HECT, UBA And WWE Domain Containing E3 Ubiquitin Protein Ligase 1 | Negative regulation contributes to the proliferation of CC.  [[63]](https://www.ncbi.nlm.nih.gov/pmc/articles/PMC8253557/) | ENSG00000277801, NORAD, MIR210HG, ENSG00000266340, ENSG00000258017, ENSG00000259865, MIR205HG, OIP5-AS1, FRMD6-AS1, ENSG00000279364, ENSG00000269958, ENSG00000279605 |
| LRP11 | LDL Receptor Related Protein 11 | It acts on proliferation, migration and invasion. It is highly expressed in high-grade squamous intraepithelial lesions and is correlated with the overall survival rate.  [[64]](https://www.ncbi.nlm.nih.gov/pmc/articles/PMC6719843/) | ENSG00000276570 |
| NUDT21 | Nudix Hydrolase 21 | It is a tumor suppressor to inhibit the progression of CC.  [[65]](https://www.nature.com/articles/s41388-021-01693-w) | ENSG00000277978, SNHG8 |
| PRKDC | Protein Kinase, DNA-Activated, Catalytic Subunit | Associated with a high mutation load in CC.  [[66]](https://pubmed.ncbi.nlm.nih.gov/32238472/) | ENSG00000257553, ENSG00000273449, MIR210HG, ENSG00000244151, ENSG00000258017, LINC00189, OIP5-AS1, ENSG00000269680, FRMD6-AS1, ENSG00000279364 |
| VIRMA | Vir Like M6A Methyltransferase Associated | It has tumor-promoting effects.  [[67]](https://www.nature.com/articles/s41598-022-11415-1) | PITPNA-AS1, ENSG00000267523, ENSG00000273449, ENSG00000279753, MIR210HG, ENSG00000272696, LINC00189, MIR205HG, OIP5-AS1, ENSG00000279364 |
| [YTHDF2](http://www.ncbi.nlm.nih.gov/gene?term=(YTHDF2%5BGene%2FProtein%20Name%5D)%20AND%20Homo%20sapiens%5BOrganism%5D) | YTH N6-methyladenosine RNA binding protein F2 | Expression is up-regulated in CC tissues. Low expression can restrict proliferation, promote apoptosis and arrest cells in S phase in cervical cancer cells. [[68]](https://link.springer.com/article/10.1186/s12935-021-01807-0) | ILRUN-AS1, SNHG8, ENSG00000279605 |
