# Supplementary material for: Upregulation of long non-coding RNA ENSG00000267838 is related to the high risk of progression and non-response to chemoradiotherapy treatment for cervical cancer
Source: Noncoding RNA Res. 2024 Oct 24;11:104–14. doi: 10.1016/j.ncrna.2024.10.004 (PMC11683307; doi:10.1016/j.ncrna.2024.10.004)
Supplement: Multimedia component 4 [file mmc4.docx]

**Supplementary Table 4 – miRNAs associated with the 49 DEGs.** Identification of miRNAs, their function in CC and their interactions with the 49 selected lncRNAs localized by AnnoLnc2.

| miRNAs | Function in the CC | LncRNAs of interaction |
| --- | --- | --- |
| miR-10-5p | Inhibition of cancer cell viability. Induction of cell cycle arrest.  [[69]](https://doi.org/10.1080/1061186X.2018.1505894) [[70]](https://www.ncbi.nlm.nih.gov/pmc/articles/PMC5740563/) | PITPNA-AS1, CDKN2B-AS1, ENSG00000244151, MIR210HG, ENSG00000257379, ENSG00000258017, ENSG00000259972, ENSG00000265401, ENSG00000266340, ENSG00000267838, ENSG00000269243, ENSG00000272234, TRIM8-DT, ENSG00000276570, ENSG00000279605. |
| miR-122-5p | Modulation of radiosensitivity.  [[71]](https://doi.org/10.1016/j.bbrc.2019.04.102) [[72]](https://pubmed.ncbi.nlm.nih.gov/31505105/) | LINC00189, MIR205HG, CDKN2B-AS1, NORAD, ENSG00000261061, ENSG00000266340, ENSG00000267815, ENSG00000269243, ILRUN-AS1, ENSG00000272696, ENSG00000276570, ENSG00000277978, ENSG00000280027. |
| miR-124-3p | Suppresses proliferation, migration and invasion of CC cells.  [[73]](https://www.ncbi.nlm.nih.gov/pmc/articles/PMC5774538/) | LINC00189, CDKN2B-AS1, MALAT1,  ENSG00000265401, ENSG00000266340, ENSG00000267838, ENSG00000269243, ILRUN-AS1, ENSG00000272696, TRIM8-DT, ENSG00000280064. |
| miR-128-3p | Inhibition of proliferation, migration and invasion of CC cells.  [[74]](https://www.tandfonline.com/doi/full/10.2147/CMAR.S265545) [[75]](https://www.spandidos-publications.com/10.3892/or.2021.8013) | ENSG00000244151, SPCS3-AS1,  ENSG00000257663, CHMP1B-AS1, ENSG00000267838, ENSG00000269958, ENSG00000272696, ENSG00000273149, ENSG00000277801. |
| miR-129-5p | Inhibition of the effects of CC cell migration and invasion.  [[76]](https://www.ncbi.nlm.nih.gov/pmc/articles/PMC6301807/) | OIP5-AS1, PITPNA-AS1, ENSG00000258232, ENSG00000259972, ENSG00000261061, ENSG00000264577, CHMP1B-AS1, ENSG00000267523, ENSG00000272234, TRIM8-DT, ENSG00000273149, ENSG00000273449, ENSG00000279364, ENSG00000279753, ENSG00000280027. |
| miR-140-3p | Prevents the proliferation of human CC cells.  [[77]](https://doi.org/10.1016/j.bmc.2019.115283) [[78]](https://www.ncbi.nlm.nih.gov/pmc/articles/PMC5356564/) | ENSG00000257553, ENSG00000258017, ENSG00000258232, SPINT1-AS1, ENSG00000277801. |
| miR-140-5p | The level of expression increases/decreases cell proliferation, migration and invasion in CC cells.  [[79]](https://www.ncbi.nlm.nih.gov/pmc/articles/PMC5356564/) [[80]](https://link.springer.com/article/10.1186/s12935-019-0744-y) | ENSG00000257553, ENSG00000266340, ENSG00000269680. |
| miR-143-3p | Suppresses the occurrence and development of CC.  [[81]](https://link.springer.com/article/10.1007/s12094-021-02687-6) | ENSG00000277801, ENSG00000279753. |
| miR-145-5p | Regulates proliferation, migration and invasion of CC cells.  [[82]](https://doi.org/10.2147/OTT.S241366) | PITPNA-AS1. |
| miR-150-5p | Promotes proliferation and EMT of CC cells.  [[83]](https://pubmed.ncbi.nlm.nih.gov/30679084/) | ENSG00000257553, ENSG00000257663, ENSG00000272234. |
| miR-182-5p | Tumor suppressor reported in CC.  [[84]](https://doi.org/10.1016/j.prp.2020.152994) | SPINT1-AS1. |
| miR-214-5p | Involved in radioresistance in the CC.  [[85]](https://advances.umw.edu.pl/en/ahead-of-print/166673/) | ENSG00000277978. |
| miR-375 | Positive regulation influences acquired resistance to paclitaxel in CC. Negative regulation involved in pelvic lymph node metastasis and FIGO stage.  [[86]](https://www.sciencedirect.com/science/article/pii/S0002944011007590) [[87]](https://journals.plos.org/plosone/article?id=10.1371/journal.pone.0109299) [[88]](https://www.nature.com/articles/bjc2013308) [[89]](https://ijgc.bmj.com/content/26/5/851) | LINC00189, SPCS3-AS1, ENSG00000257553, ENSG00000258232, SPINT1-AS1, CHMP1B-AS1, ILRUN-AS1, ENSG00000272696, ENSG00000273149, ENSG00000273449, FRMD6-AS1, OIP5-AS1. |
